# Complexity in simulation-based education: exploring the role of hindsight bias

**DOI:** 10.1186/s41077-015-0005-7

**Published:** 2016-01-11

**Authors:** Al Motavalli, Debra Nestel

**Affiliations:** 1 Department of Anaesthesia, The Royal Victorian Eye & Ear Hospital, 32 Gisborne St, East Melbourne, VIC 3002 Australia; 2 HealthPEER, Faculty of Medicine, Nursing and Health Sciences, Monash University, Melbourne, VIC Australia

**Keywords:** Cognitive Bias, Simulation Design, Hindsight Bias, Learner Reflection, Accident Investigation

## Abstract

Simulation-based education (SBE) has the potential to misrepresent clinical practice as relatively simplistic, and as being made safer through simplistic behavioural explanations. This review provides an overview of a well-documented and robust psychological construct - *hindsight bias* in the context of learning in healthcare simulations. Motivating this review are our observations that post-simulation debriefings may be oversimplified and *biased* by knowledge of scenario outcomes. Sometimes only limited consideration is given to issues that might be relevant to management in the complexity and uncertainty of real clinical practice. We use literature on *hindsight bias* to define the concept, inputs and implications. We offer examples from SBE where hindsight bias may occur and propose suggestions for mitigation. Influences of hindsight biases on SBE should be addressed by future studies.

## Background

*Hindsight bias* can be defined as the tendency to overestimate the foreseeability, inevitability, or likelihood of outcomes after they become manifest and known [1, 2]. This can systematically influence perceptions of past events [1, 2], and therefore potentially the processes of reflection, debriefing, and learning. Debriefing that includes a deliberate re-examination of simulation experiences is established practice [3–6], and studies have investigated methods of promoting learners’ reflections [7, 8]. However, the potential influences of hindsight bias on the nature of reflective processes in this context are unknown, as there is a gap in the literature exploring this issue [9]. The purpose of this commentary is to review hindsight bias in simulation-based education (SBE).

Using a vignette, the review explores our notion that debriefing is sometimes oversimplified and biased by outcome knowledge. We have observed that on occasion only limited consideration is given to potentially relevant issues related to the complexities of clinical practice. Often entailing multiple, dynamic, and sometimes conflicting demands and goals, clinical practice is complex. Such demands and goals are context-dependent, and exist under conditions of variable time pressure. We describe features of the hindsight bias literature, including the broad empirical support base, and the types of populations and contexts where hindsight bias has been investigated. The potential relevance and implications of hindsight bias to SBE are then examined and strategies to mitigate their impact offered.

### Simulation-based education for complex clinical practice

When conducting or observing debriefings, there are occasions where it might be suitable to explore a broad range of factors related to how and why learners managed a simulation scenario the way they did, in the face of uncertainty and complexity. After all, management in the context of uncertainty and complexity is surely an indisputable feature of clinical practice. However, it is our experience that when examining past simulation events (debriefing), there is a tendency for facilitators and learners to construct and accept very simplified explanations for actions and occurrences. These explanations seem to fail to account for the complexity encountered by participants in the simulation.

The vignette in Box 1 is used to illustrate relevant concepts. The situation is likely to resonate with many facilitators during debriefing. A timely call for assistance can have significant implications to patient morbidity and mortality, and may therefore be considered an important learning point [10]. As learners demonstrate this step in subsequent simulations, often immediately prompted by any sign of patient deterioration, it is reasonable to think that this learning point was successfully processed. The decision to call for help is likely to be embedded within a range of potential concerns faced by practitioners in the moment during a clinical situation. These may include assessing and managing the patient’s immediate condition, resolving diagnostic dilemmas, prioritising tasks and deciding when and where to allocate scarce human resources. Further, they are to be accomplished when the trajectory of the patient’s progress is uncertain and *unknown*.

**Box 1:** Vignette of an inter-professional simulation

An inter-professional high-fidelity crisis simulation session was conducted with a group of senior medical and nursing students.
The learning objectives were to: • Demonstrate management of a deteriorating ward patient • Demonstrate advanced life support • Demonstrate crisis resource management skills
The scenario involved a ward patient who is complaining of nausea and dizziness, but with normal vital signs. This was initially conducted with one medical and one nursing student.
During the first few minutes of the simulation, the medical student carried out initial diagnostic and therapeutic interventions with the assistance of the nursing student.
When the patient’s blood pressure dropped mildly, the students made intensive efforts to quickly establish intravenous access and set up intravenous fluids.
The medical student stated that he was unsure about the diagnosis and he re-examined the patient. The patient’s blood pressure briefly improved, but then rapidly declined and he progressed to cardiac arrest.
After the cardiac arrest was diagnosed, the medical student requested that the nursing student leave to call for help.
Resuscitation activities initially appeared to be very challenging in the face of limited human resources.
During debriefing in a separate room with all students present and watching, the facilitator used video playback to replay the scenario up to and including the initial cardiac arrest management.
The facilitator asked the lead medical student participant to discuss their experience. The student stated that he should have requested for extra assistance much earlier, as this would have helped with the arrest resuscitation.
The facilitator agreed, and in the subsequent discussion, several of the other simulation participants stated that they would call for help early in future.

In the face of such a situation, the judgement of when to relinquish immediately valuable and limited human resources to call for help is unlikely to be a clear and easy one. A learner who is overwhelmed with negotiating competing demands and goals might even overlook the decision-making step of seeking extra assistance for a prolonged period of time. This picture of context-specific complexity contrasts sharply with that of relative simplicity made with hindsight during the debriefing described in our vignette.

In light of knowledge that the patient progressed to cardiac arrest, it is possible to look back at the simulation and conclude that calling for help early was obvious and would have avoided challenges encountered with the arrest resuscitation. It might also seem that this learning point is simply and easily transferable to the management of future situations in practice. These conclusions, however, fail to address the practical issue of how the participants are to go about recognising and resolving this decision-making step amongst a number of other potential issues, particularly when the patient’s progress will *not* be readily foreseeable.

Debriefing discourse of learners and facilitators often simplifies the examination of past events, and in a way that is influenced by the knowledge of actual outcomes of the simulation. Specifically, this simplification leads to appraisals of actions made in light of simulation outcomes, even though these outcomes may not have necessarily eventuated from the same set of antecedent events, and could not have necessarily been foreseeable by learners within the simulation. In the vignette, the patient condition may not have progressed to cardiac arrest, and intense management efforts made by the two learners may have delayed or even prevented deterioration. From this perspective, which does not assume the inevitability of the patient progressing to cardiac arrest, the decision of when to call for help becomes less obvious. By calling for help early in future simulations, participants might be demonstrating that they have learned management for simulation rather than for clinical practice.

If simplification as described earlier might limit the learning that occurs for complex clinical practice, then this may have consequences for patient management and safety. *Why is it that simplification occurs and is evident in both facilitators and learners?* Fenwick and Dahlgren have recently noted limitations in SBE highlighting *complexity theory* as a means to “make visible important material dynamics, and their problematic consequences, that are not often *noticed* in simulated experiences…”(Fenwick & Dahlgren 2015, p. 359; emphasis added) [11]. That is, they acknowledge not only the interpersonal relationships of those involved in the simulation but confer at least equal power on the environment and other artefacts shaping behaviours. We propose *hindsight bias* as one possible answer to this question.

### Review strategy

We searched the databases of PsychINFO, PubMed, ERIC, CINAHL plus, Web of Science, and SCOPUS using the terms: ‘hindsight bias’ AND ‘simulation’ – no articles dedicated to exploring hindsight bias in SBE were found. The same databases were searched with the terms: ‘hindsight bias’ AND ‘reflection’ – only one relevant empirical study was found. The same databases were then searched with the term: ‘hindsight bias’, which generated 763 unique articles. Of these, two recent articles had an explicit aim to review and synthesise the hindsight bias literature [2, 12]. These were selected for outlining definitions, inputs and implications of hindsight bias. Further details on selected topics were found by searching for articles with: ‘specific term’ (e.g., video) AND ‘hindsight bias’ in the same databases. Additionally, the references of articles were used to identify relevant articles. All databases were searched on September 1 2014. Cognitive psychology books were also used.

### Cognitive biases and hindsight bias: definitions and consequences

Cognitive psychology describes cognitive biases as mental processes, that lead to systematic deviations in judgement from a norm [13]. A norm in this case, *“reflects what should be the outcome of a task carried out rationally or in a manner suitable to the situation at hand”* (Caverni et al. 1990, p.8) [13]. So using the vignette, an assertion could be made that a ‘rational’ and ‘suitable’ re-examination of the simulation includes an articulation and exploration of several factors and conditions related to management in the face of complexity and uncertainty as experienced by practitioners in the moment. This would contrast with a re-examination that offers a single cause and effect explanation or one that supports a potentially unforeseeable outcome.

Cognitive biases are considered to be unconscious, automatic, mental processing strategies, which have evolved as adaptive mechanisms to simplify complex information inputs, yet as a result, lead to biased judgements and inferences [14]. Additionally, individuals lack awareness for how these biases might impact their perception. That is, they do not realise that their judgements are biased [15]. Hindsight bias belongs to a group of biases where simplification involves a mental emphasis on factors that confirm or support a known outcome, while diminishing the significance of those identifiable factors that may favour potentially different or even contradictory outcomes [12]. Biases clearly do not prevent valuable reflective judgements and learning processes from occurring. The key issue is that with the tendency for *simplification* of this nature, the judgements and inferences are necessarily limited and biased. In SBE, this might have implications for what aspects of the simulation facilitators and learners *notice* and focus on, and hence possibly the scope and nature of learning opportunities that might be derived from reflecting.

In their review of the hindsight bias literature, Roese and Vohs summarised two important consequences of hindsight bias [2].

1. The first consequence is the construction and focus on unitary or limited causal narratives for explaining past events in relation to known outcomes, particularly with attribution of responsibility given to those most proximal to a particular outcome [2, 16, 17]. Thus hindsight bias leads to simple causal explanations that link to a manifest outcome, even though such explanations may be flawed [2]. Some empirical studies demonstrate hindsight bias occurrence depends on a plausible cause being identified and linked to an outcome [18, 19]. This feature of hindsight bias resonates with our experience of the explanations of simulation events found in debriefing discourse. In the vignette, the construction and acceptance of a simple causal link between a late call for help by the lead participant, and the difficult arrest resuscitation illustrates this point.
2. The second consequence is overconfidence in analysing and performing in similar future situations [2, 20]. This is thought due to hindsight bias minimising detailed scrutiny of past personal performances, leading to a false sense of heightened self-belief for future performance [2]. It may lead to a failure to appreciate and address the breadth and depth of factors at play when making similar future decisions – with potentially hazardous consequences. Using the vignette, the learners may be confident that they have learned to appropriately call for help early in future. However, they might fail to do so if they do not recognise the conditions that may or may not favour such an intervention in the clinical context. For example, a more explicit reflection upon conditions such as the concurrent presence of diagnostic uncertainty, vital sign changes and the limited number of novice practitioners, may support learners to quickly recognise similar conditions and act by calling for help early.

### Hindsight bias empirical evidence and investigation in other contexts

In contrast with other potentially relevant cognitive bias constructs, an extensive literature base supports hindsight bias. A recent psychology review identified over 800 published articles on this bias [2]. Cognitive psychology researchers describe hindsight bias as a ‘robust’ phenomenon - that is, observable under a wide variety of experimental conditions [12, 21–23]. It has been measured and demonstrated across age groups [21, 24], and in different cultures [25, 26]. It has been investigated in a variety of domains, including legal decision-making [27], medical diagnosis and malpractice claims [28–30], forecasting in finance [31], elections [32], consumer satisfaction studies [33], and accident investigations [34]. Safety experts have frequently cited hindsight bias as a barrier to acknowledging complexity and foreseeability issues in relation to analysing accident investigations - this can lead to investigation reports that may not maximise learning to improve future safety [35–38]. In some of these contexts, implications also extend to consequences regarding the attribution of blame or negligence.

One article was found that studied the effect of hindsight bias in relation to nurses’ reflective practices. Nurses were asked to read the same written clinical vignette describing a patient’s clinical presentation, with or without a final physician statement offering a single likely patient diagnosis (either the correct diagnosis, or one from a limited set of incorrect differentials). This physician statement was considered to represent outcome knowledge. All nurses were then asked to select what they considered to be the most likely patient diagnosis from a list that included the correct diagnosis and the limited set of incorrect differentials. Although the study suggested that nurses who were provided with a final physician statement tended to exhibit hindsight bias for the offered diagnosis, whether it was correct or not, it was acknowledged that nurses might have actually supported that diagnosis because it was stated to have come from a physician.

### Hindsight bias inputs and managing its influences

A greater understanding of hindsight bias might be gained through a brief exploration of its inputs, and this might also suggest potential targets for practice and research. Cognitive and metacognitive processes, rather than motivational ones, are considered to represent the most important factors in producing and modifying hindsight bias [2, 12]. Roese & Vohs [2] categorised three main cognitive memory inputs and described one metacognitive contributor:

1. *Recollection:* If memories cannot be clearly and accurately recalled, people tend to articulate distorted ‘memories’ that link closely with known outcomes. This is considered to be a minor effect in comparison to other inputs.
2. *Belief updating:* When presented with new information, there tends to be automatic integration with existing memory structures such that memories most consistent with this information are reinforced, and less congruous ones minimised [39, 40].
3. *Sense-making*: Simple, coherent, linear explanations of outcomes in relation to antecedent events provoke a sense of clear inevitability, and increase hindsight bias [41].
4. *Metacognitive contributions*: The apparent subjective ease with which we are able to form or process a particular explanation is found to strengthen its acceptability and increase hindsight bias. This is thought to be one mechanism by which playing video representations of past events has been demonstrated to increase hindsight bias. This is thought to result from the depiction of past events in clear continuity with their outcomes.

Because it is considered an automatic and unconscious process, hindsight bias influences have proven to be extremely difficult to eliminate [42]. Simply having knowledge that hindsight bias exists has not been demonstrated to reduce it [43]. Currently, the only empirically supported strategy that has consistently demonstrated hindsight bias reduction is the use of *counterfactual explanations* [44–48]. ‘Counterfactual’ means contrary to facts [49]. This involves the deliberate formulation of explanations for how *potential* alternative outcomes could have eventuated from the same *actual* antecedent events. Conversely, it can also involve constructing explanations for how certain *actual* outcomes could have come about as a result of different *potential* antecedent events. Experts consider it effective to use at most two to three counterfactual explanations, as generating more could increase hindsight bias, by making it subjectively difficult from a metacognitive point of view [2, 50].

In the vignette, rather than simply being designated a consequence of oversight or error, the delayed call for assistance ‘outcome’ could be reformulated to be a function of diverted attention toward immediate diagnosis and management. These immediate and intense management efforts can also be articulated as having had the purpose and potential to minimise patient deterioration. This plausible counterfactual explanation offers a *potential* alternative outcome (of delaying or preventing deterioration) for the same *actual* antecedent events (initial management actions). After acknowledging this complexity and uncertainty, a discussion may then follow which includes an appraisal of the *conditions* that did or did not favour an earlier call for help, and how these might be recognised and acted upon in a timely manner in future.

### Potential implications of hindsight bias to learning and simulation practices

Experiential learning theories share learner *reflection* of an experience as a key stage of the learning process, whereby a learner needs to “…recapture, notice and re-evaluate their experience, to work with their experience, to turn it into learning" (Boud et al. 1993, p. 9) [51]. A number of complex contextual factors might interact and influence the learner’s reflective process, including, but not limited to those related to the learner, the facilitator, the simulation design and so forth. Clearly then, hindsight bias is only one such factor that might modify this process alongside others. However, despite the potential complexity related to reflective processes and judgement formation, hindsight bias does have a strong empirical basis and has been demonstrated to be *robust*. There is compelling reason to suggest that it also has relevance in SBE, even if these involve variable and unique contexts and conditions. As such, further investigation seems warranted. Additionally, unlike other disciplines, in SBE we have the opportunity to modify future simulation experiences that will then be re-examined in debriefings. This might mean that beyond introducing counterfactual explanations to the debriefing, reducing hindsight bias through elements of simulation design may be possible. Understanding hindsight bias might serve as a useful perspective to critically appraise various simulation and debriefing practices. Clearly, any modifications to practices would need to be contextualised and balanced with other goals and overriding concerns. Below we outline some considerations and theoretical strategies to mitigate the impact of hindsight bias in SBE.

### Simulation design and debrief timing

- The *pause and discuss* method of debriefing, based Schön’s *reflection-in-action* [52–54], may prove valuable. With pauses occurring prior to significant simulation outcomes manifesting, this may help with exploring and addressing immediate learner perceptions within a simulation experience by preventing belief updating and sense-making tied to particular outcomes.
- Ending a simulation scenario prior to a significant deterioration might be another useful strategy. In our vignette, designing and running subsequent simulations where patient arrest or progressive deterioration does not occur would be one way of shifting focus toward decision-making and management in conditions of uncertainty, and away from perceptions of inevitability.
- Simulations with less experienced learners may be more at risk of oversimplification during debriefing. This can be addressed in scenario design (not single cause and effect relationships), during briefing (alerting learners to multifactorial antecedents), during the simulation (a confederate may introduce complexity) and during debriefing (proposing *did you notice* questions with counterfactuals).

### Video-assisted debriefing

- Through the clear depiction of events in continuity with outcomes, video-assisted debriefing might increase hindsight bias and this could challenge the intuitive assumption made by some simulation experts that video playback “…allow[s] participants to see how they performed rather than how they thought they performed, and…help reduce hindsight bias in assessment of the scenario.” (Fanning & Gaba 2007, p. 122) [3].
- Multiple visual perspectives (display clip from different camera angles) may interrupt the apparent simple linearity by which events may appear to be linked to one another when viewing from a single point [2].
- A facilitator could replay selected clips and direct learners focus to different physical locations, events or issues that were occurring simultaneously.
- To raise awareness and test hindsight bias, a facilitator could pause a video during playback and ask learners what happened next.

### Facilitators

- Facilitators hold significant power during debriefing [55] and therefore influence what learners focus on and how they judge their performances.
- A facilitator’s understanding of events borne of hindsight might help learners to focus on areas that yield valuable learning points. The potential pitfall is that from within this perspective, there may be an inadequate exploration of a variety of learner-centred concerns encountered in simulation. This concern also extends to facilitators debriefing one another.
- Facilitators may consider the potential influence of debriefing techniques that offer or ask for simple causal explanations linking learner actions and outcomes such as, in the *advocacy and inquiry* approach, where the facilitator promotes learner reflection by stating a combination of what is termed an *advocacy* statement – that is, an observation of learner action[s] and subjective judgment of this observation, with an *inquiry* statement [56]. Although this can prompt the learner to notice elements related to the complexity of a situation, the structure of the *advocacy* statement can sometimes be one that links a learner action with a specified outcome through a *single*, plausible, explanatory narrative. This might increase hindsight bias in participants. A potential strategy is to avoid *single* explanations and introduce at least one other counterfactual in the advocacy statement.

In seeking to notice and address issues of complexity and uncertainty, research might also be directed at defining a set of relevant contextual factors or conditions related to management in complex situations. This may lead to debriefings discussing issues experienced in the moment during complex clinical practice. This might include those factors that favoured or disfavoured certain actions being taken, whether these were intended or not. Potential debriefing topics could include issues and constructs such as: managing competing goals, task prioritisation, resource allocation strategies in time-critical and/or resource-poor situations, recognising and managing cognitive overload and fixation errors. Also, potentially relevant could be sociocultural barriers to making certain decision-making steps, including perceived consequences of decisions and issues surrounding power imbalances. Of course, these could not all be addressed in the same debriefing, but across a number of sessions, and according to specific learning objectives, simulation experiences, learner populations, and importantly, learner concerns. These conceptual distinctions could be focused on during reflection, and form the basis for articulations during debriefing discourse. Consequently, this might enable learning that is more aligned to complex, context-specific, clinical practice.

## Conclusions

Clinical practice often involves complexity and uncertainty, and learning from SBE should address issues related to management in these contexts. Drawing on findings from published reviews of hindsight bias, the construct has an extensive empirical foundation, and is pervasive across a variety of contexts. During debriefings, hindsight bias might lead to oversimplification of explanations of simulation events, with a failure to acknowledge issues related to management in complex situations, as they are experienced in the moment by learners. This might influence facilitators and learners, leading to a failure to address learning opportunities that might be relevant to patient management in practice. Knowing that the bias exists has not been demonstrated to influence its effects. Given we do not know the impact of oversimplification during debriefing on subsequent practice, it is an important target for further inquiry and research, together with inputs and consequences of hindsight bias for all facets of SBE.
